# The Nsp12-coding region of type 2 PRRSV is required for viral subgenomic mRNA synthesis

**DOI:** 10.1080/22221751.2019.1679010

**Published:** 2019-10-21

**Authors:** Tong-Yun Wang, Qiong-Qiong Fang, Feng Cong, Yong-Gang Liu, Hai-Ming Wang, Hong-Liang Zhang, Zhi-Jun Tian, Yan-Dong Tang, Xue-Hui Cai

**Affiliations:** a State Key Laboratory of Veterinary Biotechnology, Harbin Veterinary Research Institute of Chinese Academy of Agricultural Sciences, Harbin, People’s Republic of China; b Guangdong Key Laboratory of Laboratory Animals, Guangdong Laboratory Animals Monitoring Institute, Guangzhou, People’s Republic of China

**Keywords:** PRRSV, Nsp12, dimer, subgenomic mRNA, RNA synthesis

## Abstract

As one of many nonstructural proteins of porcine reproductive and respiratory syndrome virus (PRRSV), nonstructural protein 12 (Nsp12) has received relatively little attention, and its role in virus replication, if any, is essentially unknown. By the application of reverse genetic manipulation of an infectious PRRSV clone, the current study is the first to demonstrate that Nsp12 is a key component of PRRSV replication. In addition, the biochemical properties of Nsp12 were evaluated, revealing that Nsp12 forms dimers when exposed to oxidative conditions. Furthermore, we systemically analyzed the function of Nsp12 in PRRSV RNA synthesis using a strand-specific PCR method. To our surprise, Nsp12 was not found to be involved in minus-strand genomic RNA (-gRNA) synthesis; importantly, our results indicate that Nsp12 is involved in the synthesis of both plus- and minus-strand subgenomic mRNAs (+sgmRNA and -sgmRNA). Finally, we found that the combination of cysteine 35 and cysteine 79 in Nsp12 is required for sgmRNA synthesis. To our knowledge, we are the first to report the biological role of Nsp12 in the PRRSV lifecycle, and we conclude that Nsp12 is involved in the synthesis of both + sgRNA and -sgRNA.

## Introduction

Porcine reproductive and respiratory syndrome virus (PRRSV), a severe pathogen belonging to the *Arteriviridae* family that affects the global swine industry, is a positive-strand RNA virus with a genome of approximately 15 kb [1]. The genomic RNA encodes RNA replicases (ORF1a and ORF1b), glycoproteins GP 2 to GP 5, the integral membrane protein M, and the nucleocapsid protein N (ORFs 2–7) [1]. ORFs 2–7 have been extensively investigated [1–3], and an increasing number of studies have focused on ORF1a and ORF1b, which encode polyproteins that are processed into smaller protein products designated as nonstructural proteins (Nsps). The roles for most Nsps in PRRSV have been explored to date, and Nsp1α/β, Nsp2, Nsp4 and Nsp11 have been implicated in modulating host immune responses to PRRSV infection [4–9]. In addition, Nsp1 function is related to subgenomic mRNA synthesis regulation [10], and Nsp2 and Nsp3 are involved in inducing replication-associated membrane rearrangement [11,12]. Moreover, the Nsp9 RNA polymerase and Nsp10 helicase are key enzymes for arterivirus RNA synthesis and are reportedly responsible for the virulence of highly pathogenic PRRSV [13]. The roles of Nsps in viral pathogenesis and host immunity are also being explored.

Although many Nsp functions have been discovered, information about the biological role of PRRSV Nsp12 is limited. Dong et al. investigated the Nsp12 interactome with cellular proteins, verified the interaction between the cellular chaperone HSP70 and Nsp12, and demonstrated that Nsp12 recruits HSP70 to maintain its own stability and promote viral replication [14]. Recently, Li et al. reported that porcine galectin-3 (GAL3) interacts with Nsp12 and showed that GAL3 overexpression significantly suppresses the replication of type 1 and 2 PRRSV strains [15]. Nsp12 was also found to induce the phosphorylation of signal transducer and activator of transcription 1 (STAT1) and the expression of proinflammatory cytokines and chemokines, including IL-1β, IL-8, chemokine ligand 2 (CCL2) and chemokine (C-X-C motif) ligand 10 (CXCL10), which may contribute to PRRSV pathogenesis [16]. However, all of these studies focused on the interaction of host proteins with Nsp12, but no information describes how Nsp12 participates in the replication phase of the PRRSV lifecycle. A recent study attempting to explore the interaction network involving most PRRSV Nsps demonstrated that Nsp12 may serve as a hub of the Nsp interactome along with Nsp9 [17]; this study also indicated that Nsp12 may be a major component of the replication and transcription complexes (RTCs) [17].

Here, we investigate the biochemical properties of Nsp12 and further identify Nsp12 as a key component of PRRSV replication. Notably, we demonstrate for the first time that Nsp12 is involved in viral subgenomic mRNA (sgmRNA) synthesis but not in minus-strand genomic RNA (-gRNA) synthesis.

## Materials and methods

### Plasmids and PRRSV infectious clones

Nsp12 was cloned from the HuN4 infectious cDNA clone and expressed as a fusion protein with an HA tag at its N-terminus using the expression vector pCMV-HA (Clontech, USA). A series of plasmids expressing Nsp12 substitution mutations was constructed. Mutagenesis PCR was used to create mutations at three cysteine sites. The C29A mutant was generated using the C29A forward primer and the C29A reverse primer, and the C35A and C79A mutants were constructed using the C35A and C79A forward and reverse primers, respectively. For site-specific mutation of the PRRSV infectious clone (PRRSV HuN4-F5) [18], the Nsp9 to Nsp12 regions were cloned into the pUC57 vector; after mutation of the indicated site, this region was religated to the infectious clone using the *NheI* and *AscI* sites. All constructed mutants were confirmed by DNA sequencing. The sequences of the PCR primers are provided in Table 1.

**Table 1.** Primers used in this study.

Primers	Nucleotide sequence (5’–3’)
F-6(-gRNA RT Primer and F Primer)	GTATAGGTGTTGGCTCTATGC
F-12(-sgRNA RT Primer and sgRNA F Primer and -gRNA F Primer)	GTGTTGGCTCTATGCCACGGC
R-343(-gRNA Nested R Primer)	ATAAAATAGACCCAGCACCCC
R-683(-gRNA R Primer)	GGAGCGGTAAGTTGGTTAACACA
R-15085(sgRNA R Primer)	CTCCACAGTGTAACTTATCCTCC
Nsp12-R(+gRNA RT Primer)	ATTCAGGCCTAAAGTTGGTTCA
F-9228(+gRNA F Primer)	ACCATCACAGACTCACCATCAT
R-9668(+gRNA R Primer)	TCGCACTCACTACAAGAACCA
F30(sgRNA Nested F Primer)	GCATTTGTATTGTCAGGAGC
R15042(sgRNA Nested R Primer)	CCAGCGCCCTGATTGAAGGC
F-9252(+gRNA Nested F Primer)	CTAGGTTGCAGGATAATAAATG
R-9606(+gRNA Nested R Primer)	GCTGGTGGAAGTGGGTGTGGTA
Nsp12-C25A-F	GTACTTGGACCCCGCGATGGGCCCTGCTC
Nsp12-C25A-R	GAGCAGGGCCCATCGCGGGGTCCAAGTAC
Nsp12-C39A-F	GGGCCCTGCTCTTGCGAACAGAAGGGTTG
Nsp12-C39A-R	CAACCCTTCTGTTCGCAAGAGCAGGGCCC
Nsp12-C79A-F	CAAAATTCTGGCGGCGGCGGAGTTCTCGC
Nsp12-C79A-R	GCGAGAACTCCGCCGCCGCCAGAATTTTG
Nsp12-Del-F	GGCCGCCATTTCACCTAGTAATAACTTGCAAG
Nsp12-Del-R	CTTGCAAGTTATTACTAGGTGAAATGGCGGCC
qPCR-F	ACCGTCGTACGTGCTGAACTG
qPCR-R	GACGACAGGCCACCTCTCTTA
qPCR probe	FAM- ACGACTTACTGGTCACGC -MGB

### Sequence alignment.

The deduced amino acid sequences of the different Nsp12 strains were examined using the ClustalW method in Lasergene software (version 7.1) (DNASTAR Inc., Madison, WI, USA). Multiple sequence alignments were conducted using GeneDoc software.

### Cells and virus stocks

MARC-145, BHK and HEK293 T cells were maintained in Dulbecco’s Modified Eagle’s Medium containing 10% FBS. The PRRSV HuN4-F5 strain is a highly pathogenic (HP-PRRSV) strain isolated from a farm in 2006 (GenBank accession no. EF635006) [19,20].

### Antibodies

An anti-HA antibody and an anti-actin antibody were purchased from Vazyme (Vazyme, China). An SR-30 FITC-conjugated mAb was purchased from Rural Technologies (Brookings, SD). Antibodies against Nsp12 were kindly provided by Professor Changjiang Weng at Harbin Veterinary Research Institute of the Chinese Academy of Agricultural Sciences [21].

### Direct immunostaining for recombinant virus rescue

MARC-145 cells were transfected with the recombinant infectious clone using X-tremeGENE HP DNA transfection reagent (Roche, Basel, Switzerland) according to the manufacturer's protocol. Seven days posttransfection, the cells were washed three times with cold PBS and then treated with 100% alcohol for 30 min. The cells were then incubated with the SR-30 FITC-conjugated N protein-specific antibody (1:1000 dilution) for 2 h at 37°C and washed three times with cold PBS; images were acquired using a fluorescence microscope.

### Transfection and Western blotting

Cells were transiently transfected with the indicated plasmids using X-tremeGENE HP DNA transfection reagent. At 48 h posttransfection (hpt), the cells were collected, washed once with PBS and lysed in RIPA Lysis Buffer containing a protease inhibitor cocktail (Roche). Dimer detection was performed as previously described [22]. Proteins in the cell lysates were separated by SDS-PAGE, transferred to PVDF membranes (Millipore, Germany) and probed with the indicated antibodies.

### Sucrose gradient centrifugation

MARC-145 cells were infected with PRRSV HuN4 at an MOI of 0.1 and maintained at 37°C and 5% CO_2_ until a 60% to 80% cytopathic effect (CPE) was reached, as described in previous publications [23]. Cell supernatants were collected and clarified at 5,000 × g for 1 h at 4°C to remove nonadherent cells and cellular debris. The supernatants were collected and pelleted twice through a 30% sucrose cushion as previously reported [22]; the purified virions were applied to 10% to 35% sucrose cushions and centrifuged. Typically, 12 fractions were collected from the top to the bottom and analyzed by Western blot.

### Genomic RNA and sgmRNA detection

BHK cells were seeded in 12-well plates; upon reaching 80% confluence, the cells were transfected with 2 μg of the indicated plasmids using X-tremeGENE HP DNA transfection reagent. Total cellular RNA was isolated from the transfected cells at 24 hpt using an RNeasy® Plus Mini Kit (QIAGEN). In addition, 2 U of RNase-free recombinant DNase I (BioLabs) was applied at 37°C for 30 min to eliminate the effect of transfected plasmid DNA, followed by the addition of 0.5 M EDTA at 75°C for 10 min to inactivate DNase I. Strand-specific PCR was used to detect -gRNA, positive-strand genomic RNA (+gRNA), positive-strand subgenomic RNA 7 (+sgmRNA7) and negative-strand subgenomic RNA 7 (-sgmRNA7) as previously described [10,24,25]. For -gRNA detection, the RT primer F-6 was used for first-strand cDNA synthesis; for + gRNA detection, the primer Nsp12-R was used for first-strand cDNA synthesis. The primer F-12 was employed for (-)sgmRNA7 first-strand cDNA synthesis, and the Oligo-dT primer was employed for (+)sgmRNA7 first-strand cDNA synthesis. The cDNA was first treated with 2 μg of RNase (TIANGEN, Beijing) at 37°C for 30 min and then at 95°C for 10 min to eliminate remaining RNA. For -gRNA, primary PCR was performed with primers F-6 and R-683 using 2× TSINGKE Master Mix (TSINGKE, Harbin). Nested PCR was conducted with the internal primer pair F12 and R-343. For + gRNA, the F-9228 and R-9668 primers were used to perform the primary PCR, and nested PCR was performed using the internal primer pair F-9252 and R-9606. For (+)sgmRNA7 detection, (+)sgmRNA7 cDNA was amplified to obtain the leader-body junction-containing fragment using the primers F12 and R-15085. Nested PCR was carried out with the internal primer pair F30 and R15042. For (-)sgmRNA7 detection, (-)sgmRNA7 cDNA was amplified using the primers F12 and R-15085 and the nested PCR pair F30 and R15042.

### Real-time quantitative PCR (RT-qPCR)

RT-qPCR was performed to quantify -gRNA and + gRNA. BHK cells were seeded in 12-well plates and transfected with 2 μg of the indicated plasmids (HuN4-F5, HuN4-F5-Del-Nsp12) using X-tremeGENE HP DNA transfection reagent upon reaching 80% confluence. Total RNA was extracted from the transfected BHK cells with TRIzol reagent (Sangon Biotech, Shanghai), and 2 μg of RNA was reverse transcribed into cDNA using oligo (dT) or F6 to detect -gRNA and + gRNA, respectively. RT-qPCR analysis of the viral genomic RNA was performed with qPCR-F, qPCR-R and the TaqMan probe (Table 1). Data were analyzed using the MxPro-Mx3005P (standalone) real-time PCR system.

## Results

### Nsp12 is critical for PRRSV replication

**Figure 1.**
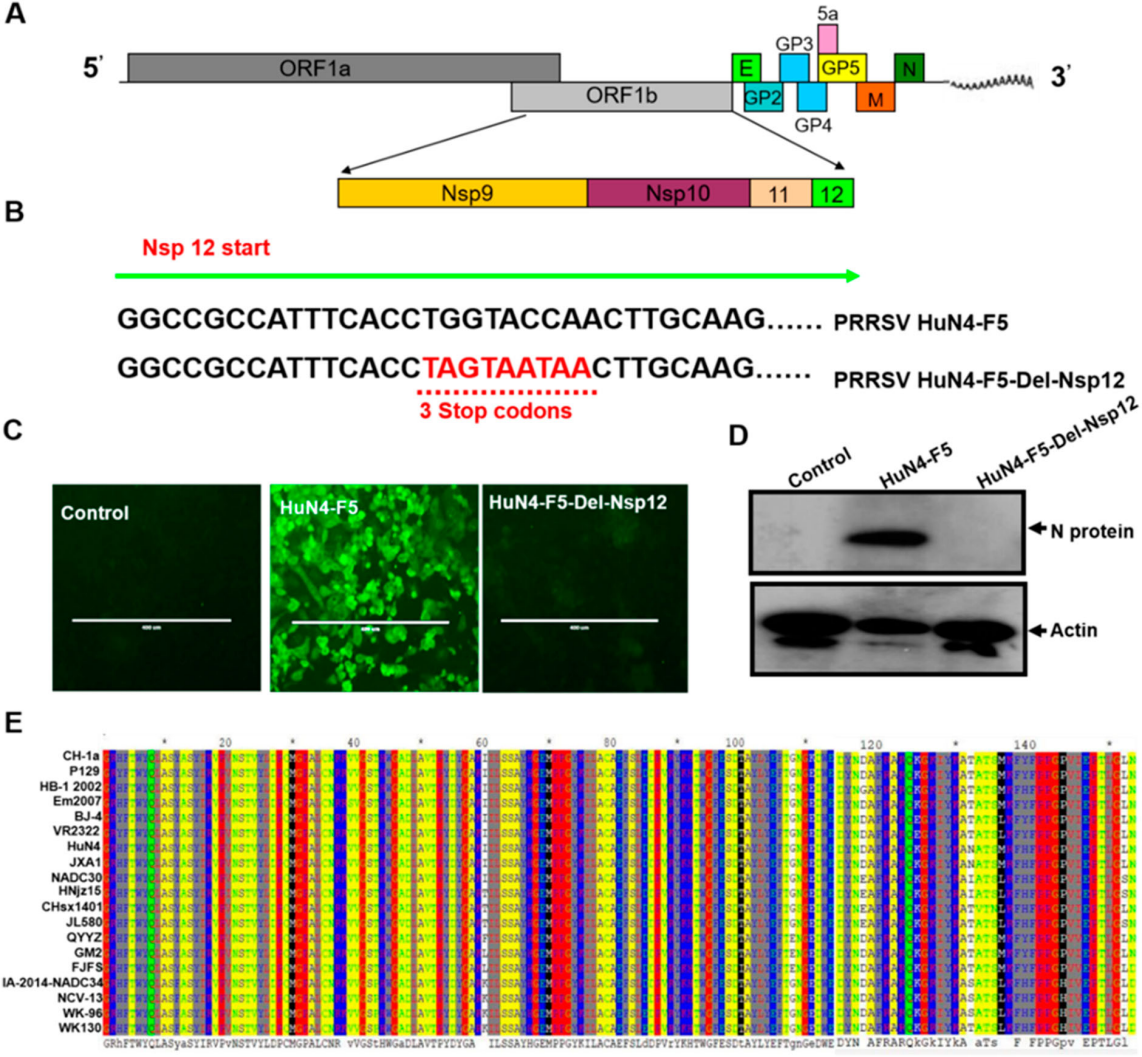
Nsp12 is required for PRRSV replication. (**A**) Schematic representation of the PRRSV genome structure. (**B**) Construction of PRRSV-HuN4-F5-del-Nsp12; the 6th-8th amino acids of Nsp12 were mutated to three consecutive stop codons (TAG and TAA). (**C**) Nsp12 is required for PRRSV replication. MARC-145 cells were transfected separately with the PRRSV-HuN4-F5 infectious clone and PRRSV-HuN4-F5-del-Nsp12. At seven days posttransfection, the transfected cells were detected using an anti-N protein antibody (green). The experiments were performed three times, and a representative result is shown. (**D**) Rescued viruses were detected by Western blotting using β-actin as an internal control. (**E**) Nsp12 amino acid sequences of different type II PRRSV strains were compared. The above experiments were performed three times, and a representative result is shown.

In this study, we used the HuN4-F5 infectious clone (a highly pathogenic PRRSV strain) as a model to explore the function of Nsp12. As shown in Figure 1A, the coding region for Nsp12 is located at the end of ORF1b, and the protein is cleaved from the ORF1ab large polyprotein by Nsp4 [26]. To assess whether Nsp12 is a key component of PRRSV replication, we introduced triple continuous stop codons into the N terminal region (6-8 amino acids) to abrogate the translation of nsp12; we designated this clone HuN4-F5-Del-Nsp12 (Figure 1B). After confirmation by DNA sequencing, we transfected both the HuN4-F5 and HuN4-F5-Del-Nsp12 infectious clones into MARC-145 cells and detected the N protein of PRRSV by immunofluorescence. The result indicated that Nsp12 is required for PRRSV replication (Figure 1C), which was confirmed by Western blotting (Figure 1D). We next determined whether Nsp12 is conserved in PRRSV by comparing the Nsp12 amino acid sequences of different type II PRRSV strains, including two classic strains, VR-2332 and CH-la, and the highly pathogenic strains HuN4, JXA1, NADC30 and NADC34. As shown in Figure 1E, Nsp12 sequences are highly conserved in PRRSV regardless of whether the strain is classic or highly pathogenic, suggesting that Nsp12 may play an important role in the PRRSV lifecycle.

### Nsp12 forms dimers under oxidizing conditions

**Figure 2.**
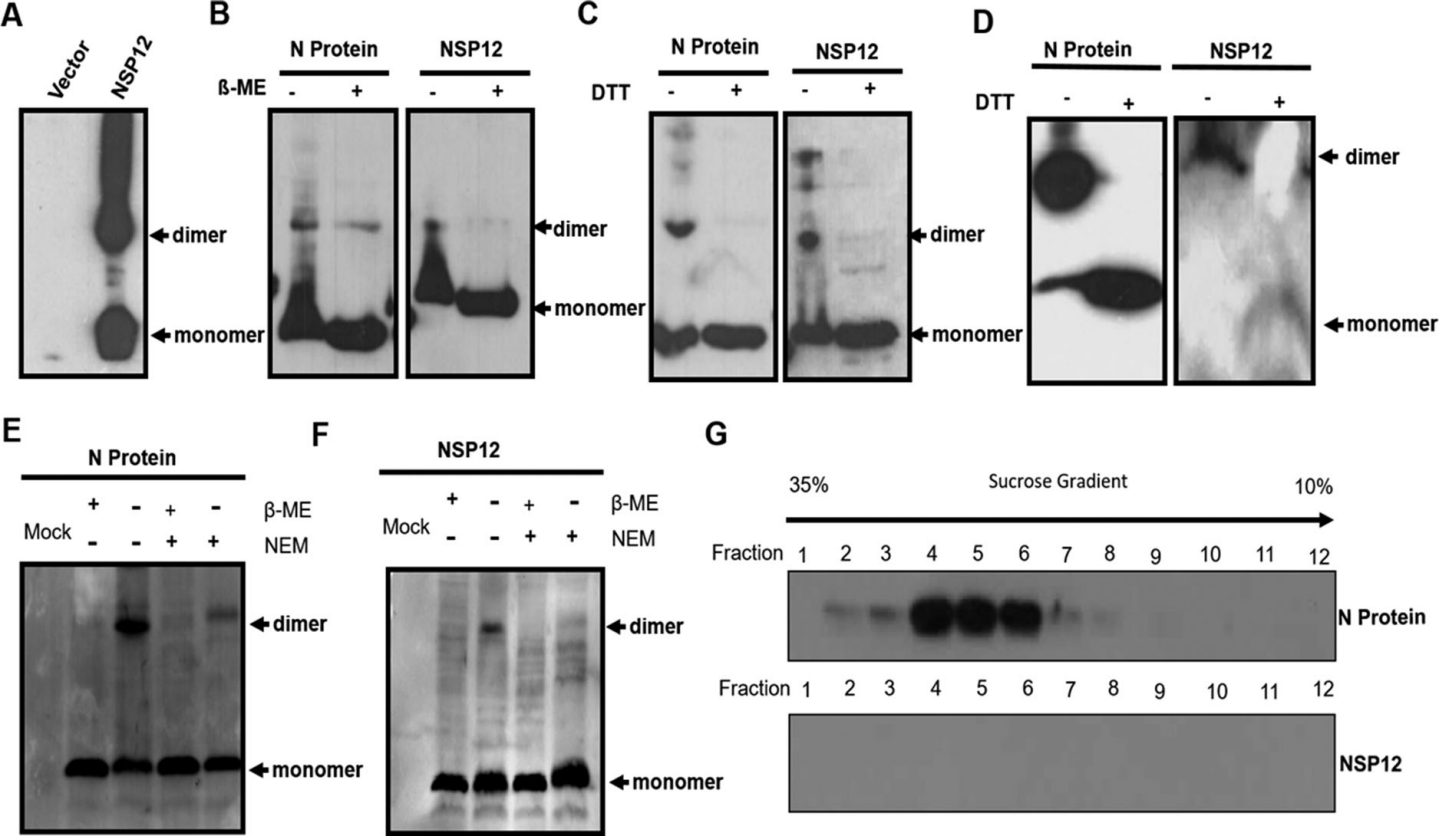
Nsp12 forms dimers under oxidizing conditions. (**A**) and (**B**) Nsp12 forms dimers, and dimer formation is mediated by covalent disulfide bonds. 2-mercaptoethanol (ß-ME) and dithiothreitol (DTT) block cysteine-mediated dimerization. The N protein was used as a positive control. (**C**) Nsp12 homodimerization due to exposure to the oxidizing extracellular environment after cell lysis. HEK293 T cells were seeded in twelve-well plates and transfected with 2 μg of pCMV-HA-N and (**D**) pCMV-HA-Nsp12 plasmids; at 24 hpt, the cells were treated with RIPA lysis buffer with or without 20 mM N-ethylmaleimide. The lysates were also treated with 4x loading buffer with or without β-ME. (**E**) Nsp12 is not incorporated into virions. MARC-145 cells were infected with PRRSV-HuN4 at an MOI of 0.01; 72 h later, the supernatants were collected and pelleted twice through a 30% sucrose cushion, after which the purified virions were fractionated using 10% to 35% sucrose cushions. Samples were collected from the top to the bottom and analyzed by Western blotting. These experiments were performed three times, and a representative result is shown.

Because Nsp12 was found to be indispensable for PRRSV replication, we sought to further analyze the biochemical properties of Nsp12. After transfection, we found that Nsp12 could form dimers and even polymers; this ability was similar to that of the N protein of PRRSV [22,27], which was used as a positive control (Figure 2A and B). However, Nsp12 dimer formation was significantly blocked when reducing agents such as dithiothreitol (DTT) and 2-mercaptoethanol (β-ME) were added (Figure 2A and B), indicating that dimerization is mediated by covalent disulfide bonds. There are three cysteines at positions 29 (Cys29), 35 (Cys35), and 79 (Cys79) of Nsp12 that may contribute to dimer formation. Thus, we replaced all of the cysteines with alanine, either singly or in combination (Fig. S1A), and found that only the triple cysteine mutation completely blocked dimer formation (Fig. S1B and C), suggesting that any cysteine in Nsp12 can form disulfide linkages.

N protein homodimerization occurs upon exposure to the oxidizing extracellular environment after cell lysis [22]; to evaluate whether Nsp12 has similar biochemical properties, transfected cells were harvested in lysis buffer in the presence or absence of 1 mM N-ethylmaleimide (NEM), a small membrane-permeable compound that can irreversibly modify the free thiols of cysteine residues, thereby preventing de novo disulfide bond formation. In the presence of NEM, we did not detect dimerization of either the N protein or Nsp12 (Figure 2C and D), indicating that Nsp12 dimerization occurs under oxidizing conditions. We next investigated whether the disulfide bonds of Nsp12 can be detected in extracellular virions, similar to the N protein [22]. To detect whether Nsp12 has the potential to incorporate into virions, purified virions were fractionated using 10% to 35% sucrose cushions, and samples were collected from the top to the bottom and analyzed by Western blot. Using the N protein as a positive control, no Nsp12 was observed in highly purified virus particles (Figure 2E). These results indicate that Nsp12 has an intrinsic ability to form disulfide bonds but only under oxidizing conditions in cell lysates.

### Nsp12 is involved in the synthesis of both plus- and minus-strand subgenomic mRNAs but not in that of genomic RNA

**Figure 3.**
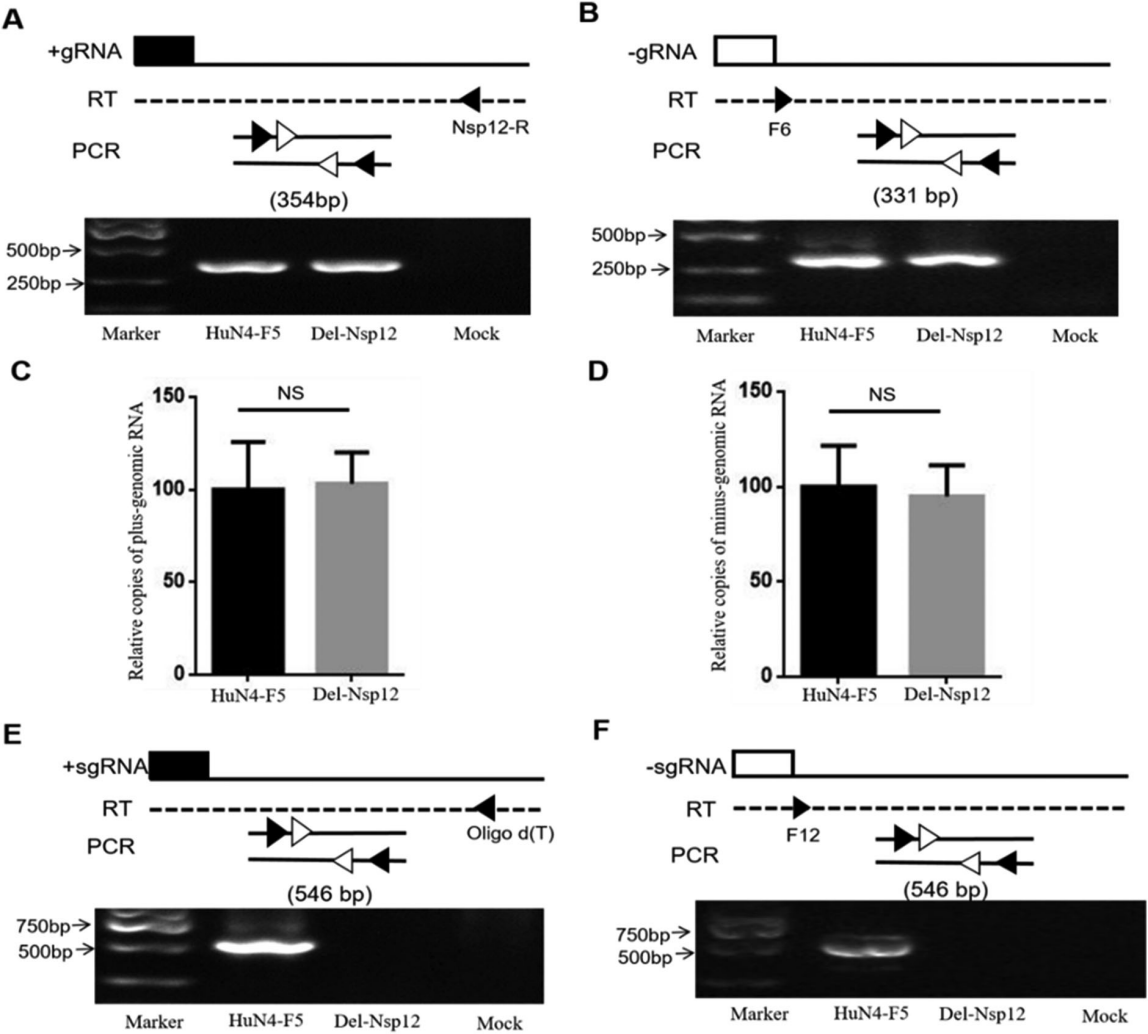
Nsp12 is involved in the synthesis of both plus- and minus-subgenomic mRNAs but not minus-genomic RNA. **(A)** A strand-specific PCR method was used to detect positive-strand and **(B)** negative-strand genomic RNA. The reverse transcription primers, PCR primers and PCR products used are presented in the upper panel. **(C)** Plus-genomic RNA and **(D)** minus-genomic RNA were quantified by qPCR. Copies of PRRSV-HuN4-F5 were set as 100%. NS stand for *p* > 0.05. **(E)** HuN4-F5-Del-Nsp12 plus-subgenomic mRNA and **(F)** minus-subgenomic mRNA synthesis was blocked by Nsp12 deletion. These experiments were performed three times, and a representative result is shown.

Nsp12 is encoded by ORF1b, and this region is recognized as being correlated with viral RNA synthesis. Therefore, we hypothesized that Nsp12 may be involved in viral -gRNA synthesis. The PRRSV infectious clone used in this study is very large and difficult to transfect. Because the transfection efficacy of BHK cells was higher than that of MARC145 cells, we used the former cells in this study. To investigate the involvement of Nsp12 in viral gRNA synthesis, we transfected BHK cells with HuN4-F5 and HuN4-F5-Del-Nsp12, respectively, isolated total cellular RNA at 24 hpt, and used a strand-specific PCR method to detect + gRNA and -gRNA as previously described [24,25]. The reverse transcription primers, PCR primers and PCR products are depicted in Figure 3 (upper panel). To our surprise, the + gRNA and -gRNA of HuN4-F5-Del-Nsp12 was not affected (Figure 3A and B). To confirm this, we further detected gRNA by qPCR and obtained similar results (Figure 3C and D). We next examined whether + sgmRNA and -sgmRNA were affected, and the results showed that both + sgmRNA and -sgmRNA were abolished when Nsp12 was inactivated (Figure 3E and F). The above data demonstrate that Nsp12 is involved in viral sgmRNA synthesis but not in gRNA synthesis.

### The combination of C35 and C79 in Nsp12 is crucial for PRRSV sgmRNA synthesis

**Figure 4.**
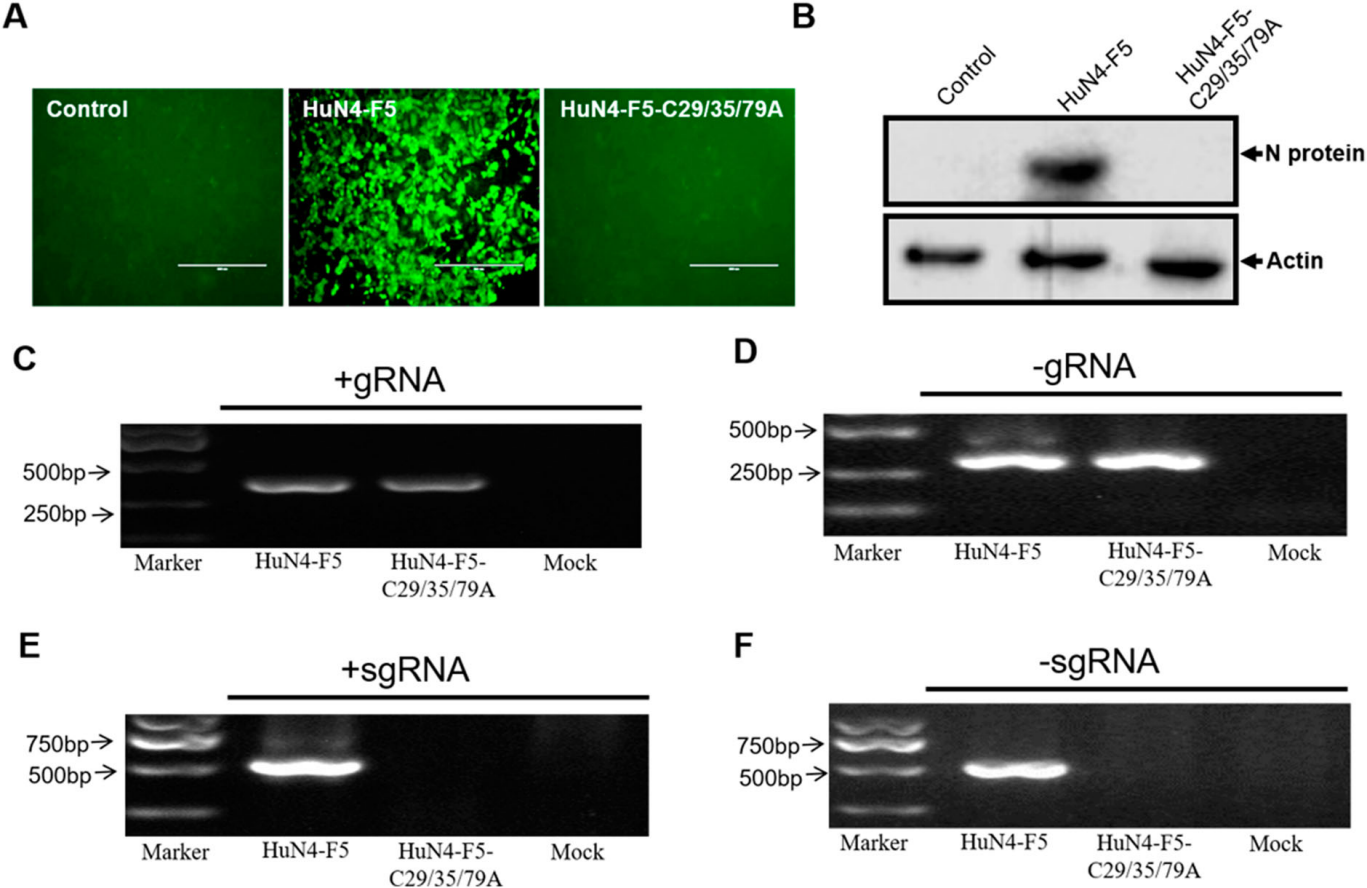
Three conserved cysteines of Nsp12 are involved in sgmRNA synthesis. (**A**) Three cysteines are essential for PRRSV replication. MARC-145 cells were transfected separately with the PRRSV-HuN4-F5 infectious clone and HuN4-F5-C29/35/79A. At seven days posttransfection, rescued viruses were detected using an anti-N protein antibody (green). (**B**) The rescued viruses were detected by Western blotting using β-actin as an internal control. (**C**) Positive-strand and (**D**) negative-strand genomic RNA synthesis was not influenced by HuN4-C29/35/79A. (**E**) Positive-strand and (**F**) negative-strand subgenomic RNA synthesis was blocked by HuN4-F5-C29/35/79A. These experiments were performed three times, and a representative result is shown.

**Figure 5.**
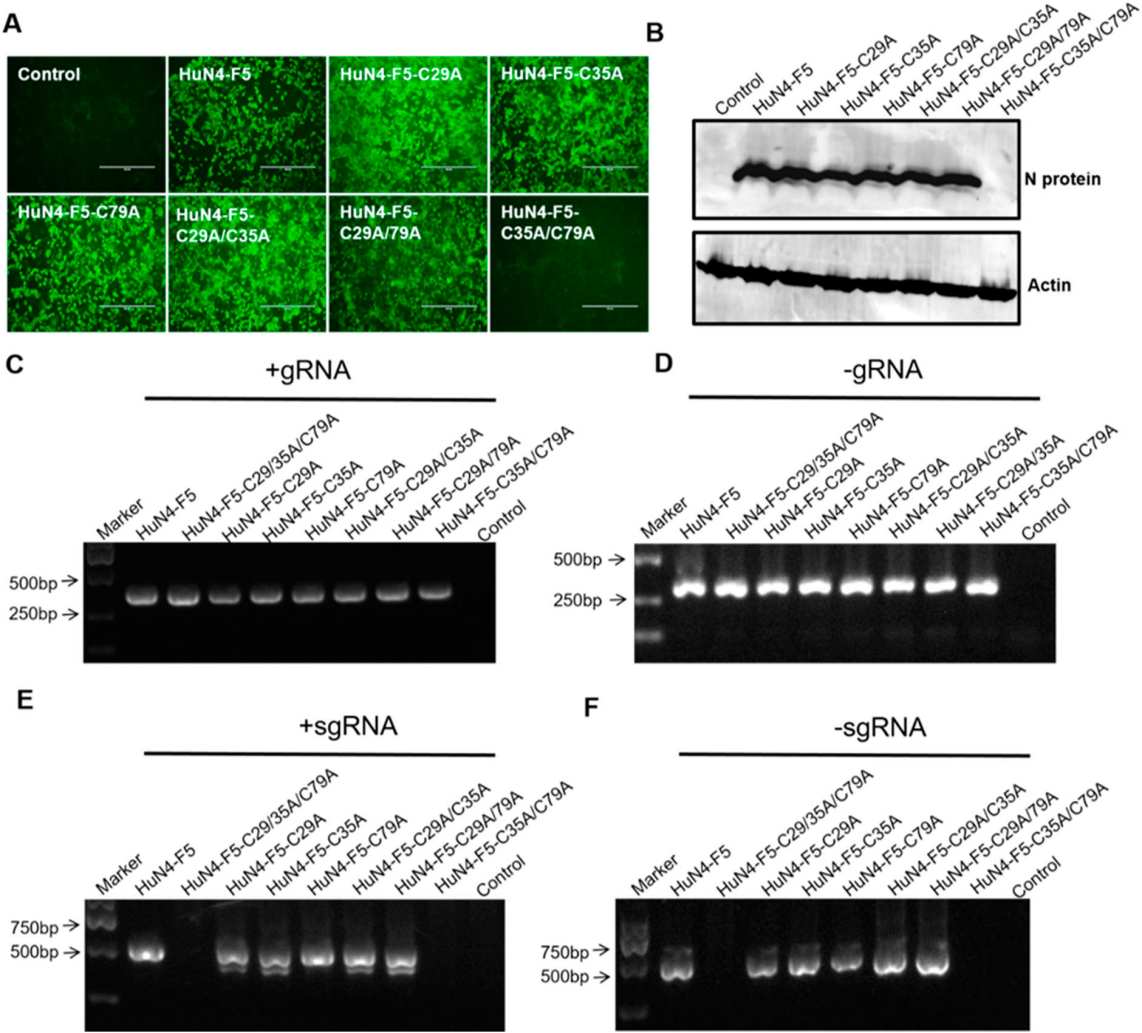
The combination of C35 and C79 in Nsp12 is involved in PRRSV sgmRNA synthesis. (**A**) C35 and C79 are involved in for PRRSV replication. MARC-145 cells were transfected separately with the PRRSV-HuN4-F5 infectious clone and indicated clones. At seven days posttransfection, rescued viruses were detected using an anti-N protein antibody (green). (**B**) The rescued viruses were detected by Western blotting using β-actin as an internal control. (**C**) Positive-strand and (**D**) negative-strand genomic RNA synthesis was not influenced by cysteine mutations. (**E**) Positive-strand and (**F**) negative-strand subgenomic RNA synthesis was blocked by HuN4-F5-C35/79A. These experiments were performed three times, and a representative result is shown.

Because Nsp12 is involved in viral sgmRNA synthesis, we further sought to determine whether the three cysteines of Nsp12 are involved in sgmRNA synthesis (the three mutations are illustrated in Fig. S1A). We replaced three cysteines in the PRRSV HuN4 infectious clone with alanine by reverse genetic manipulation to produce PRRSV HuN4-F5-C29/35/79A and found that these three cysteines were critical for PRRSV replication (Figure 4A and B). Strand-specific PCR analysis indicated that -gRNA was not affected (Figure 4C and D) but that the sgmRNA synthesis of HuN4-F5-C29/35/79A was completely blocked (Figure 4E and F). We next assessed whether single and double cysteine mutations affected viral replication by constructing six mutant clones: HuN4-F5-C29A, HuN4-F5-C35A, HuN4-F5-C79A, HuN4-F5-C29/35A, HuN4-F5-C29/79A and HuN4-F5-C35/79A. According to the results, the single cysteine mutations (C29A, C35A and C79A) successfully rescued the virus, as did the double cysteine mutations C29/35A and C29/79A (Figure 5A and B). However, to our surprise, we failed to rescue the virus with the recombinant clone HuN4-F5-C35/79A (Figure 5A and B), indicating that the combination of C35 and C79 in Nsp12 is critical for PRRSV replication. Further analysis indicated that the sgmRNA synthesis of HuN4-F5-C35/79A was also blocked. Thus, the combination of C35 and C79 in Nsp12 is crucial for PRRSV sgmRNA synthesis (Figure 5E and F).

## Discussion

In this study, we first show that Nsp12 is essential for PRRSV replication, and we also demonstrate that Nsp12 can form dimers when exposed to oxidizing conditions. Dimerization is sometimes a requirement for the biological functions of viral proteins [28–31]. Here, we report that Nsp12 has an intrinsic ability to form disulfide bonds in cell lysates but only under oxidizing conditions; thus, Nsp12 dimerization is not required for PRRSV replication, potentially constituting a situation similar to that of the N protein of PRRSV [22].

This study is also the first to find that Nsp12 is not involved in gRNA synthesis but is involved in the synthesis of sgmRNA. Arterivirus RNA synthesis requires the formation of double-membrane vesicles (DMVs) [12,32], which provide replication sites for RTC formation [33,34]. It has been proposed that Nsp2, Nsp3 and Nsp5 of arteriviruses participate in modulating membrane curvature and DMV formation [12,35]. PRRSV is closely related to EAV, and Nsp2, Nsp3, and Nsp5 of PRRSV may also have the same roles in DMV formation [17]. As the arterivirus RNA polymerase, Nsp9 has been recognized as a core component of RTCs, and PRRSV Nsp9 plays a central role in the RTC machinery and interacts with the N protein [36], Nsp7α [37], Nsp1α, Nsp1β, Nsp3, Nsp7β, Nsp8, Nsp11, and Nsp12 [17]. Furthermore, RNA synthesis efficiency correlates closely with Nsp9 and consistently with PRRSV virulence [13,38,39]. Nonetheless, arterivirus sgmRNA synthesis depends on the Nsp1 autoprotease [10,40,41]. PRRSV Nsp1α reportedly binds directly to Nsp9 [17], and EAV Nsp1 controls the accumulation of full-length and subgenome-length minus-strand templates for viral mRNA synthesis [42]. We speculate that the component necessary for PRRSV gRNA synthesis differs from that required for sgmRNA synthesis, which may be the reason that gRNA is not influenced by Nsp12. However, further exploration is needed.

In the current study, we also demonstrated for the first time that Nsp12 is involved in sgmRNA synthesis, illustrating that the combination of C35 and C79 of Nsp12 is essential for PRRSV replication. We speculate that C35 and C79 of Nsp12 may be located at an interface motif that interacts with other proteins (including viral or host proteins) critical for PRRSV sgmRNA synthesis, whereby double mutation in this region abolishes these interactions. Similar to the lack of a direct association between the RNA helicase (Nsp10) and Nsp9, there may be indirect interaction of PRRSV Nsp10 with Nsp9 via Nsp12 [17]. A recent study demonstrated multiple interactions of Nsp12 with viral Nsps, suggesting that Nsp12 likely plays an important role in the viral RNA synthesis process as a crucial component of the RTC. Furthermore, double mutation of the two cysteines may also directly influence the folding and function of nsp12 even though the two residues are not on the protein surface.

## Supplementary Material

Supplemental Material
