# Supplementary material for: The Nsp12-coding region of type 2 PRRSV is required for viral subgenomic mRNA synthesis
Source: Emerg Microbes Infect. 2019 Oct 21;8(1):1501–10. doi: 10.1080/22221751.2019.1679010 (PMC6818116; doi:10.1080/22221751.2019.1679010)
Supplement: Supplemental Material [file TEMI_A_1679010_SM4187.zip › Figure_S_legend_final_final.docx]

**FIG. S Any cysteine in Nsp12 can form disulfide linkages.** (**A**) Schematic representation of the replacement of cysteines with alanine, either singly or in combination. (**B**) and (**C**) Only the triple cysteine mutation completely blocked dimer formation. The above experiments were performed three times, and a representative result is shown.
