# Supplementary figures and images for: The Nsp12-coding region of type 2 PRRSV is required for viral subgenomic mRNA synthesis
Source: Emerg Microbes Infect. 2019 Oct 21;8(1):1501–10. doi: 10.1080/22221751.2019.1679010 (PMC6818116; doi:10.1080/22221751.2019.1679010)

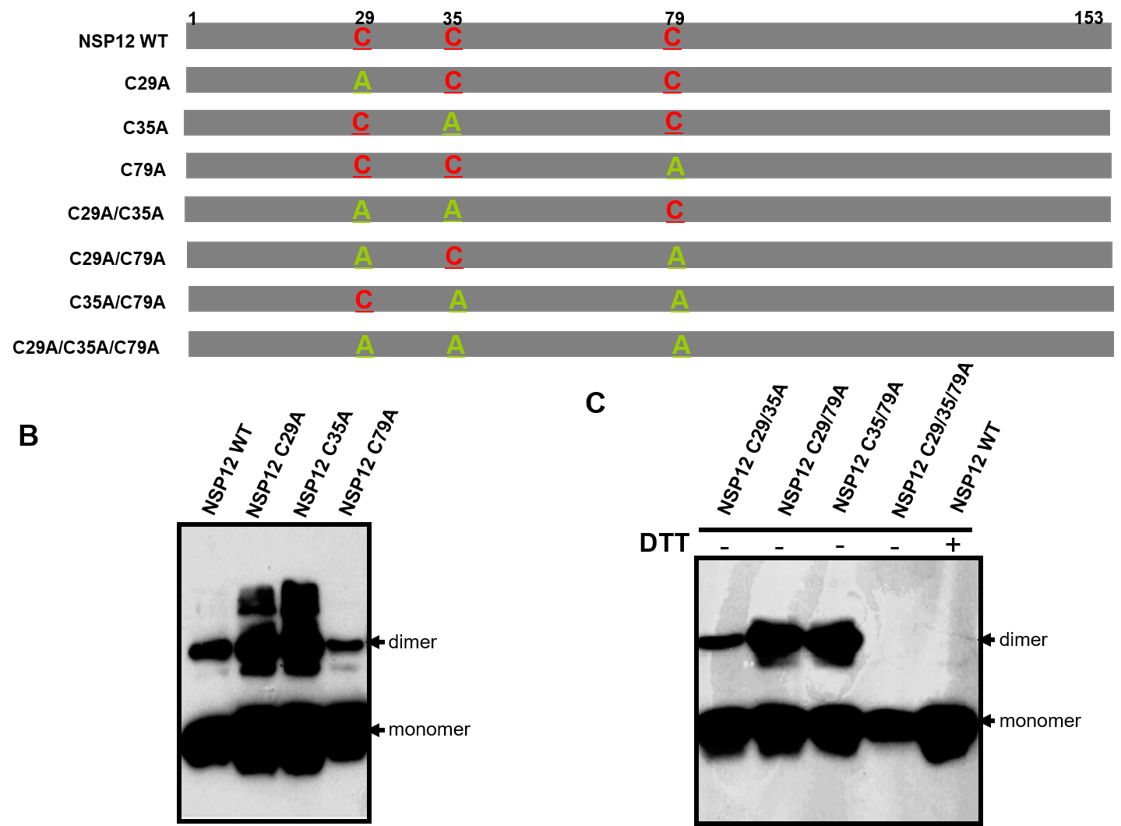

Supplement: Supplemental Material [file TEMI_A_1679010_SM4187.zip › Figure supplementary.tif]
